# Dataset on absorption spectra and bulb concentration of phenolic compounds that may interfere with onion pyruvate determinations

**DOI:** 10.1016/j.dib.2017.01.015

**Published:** 2017-02-07

**Authors:** Vanesa H. Beretta, Florencia Bannoud, Marina Insani, Claudio R. Galmarini, Pablo F. Cavagnaro

**Affiliations:** a Consejo Nacional de Investigaciones Científicas y Técnicas (CONICET), Av. Rivadavia 1917, Buenos Aires C1033AAJ, Argentina; b Instituto Nacional de Tecnología Agropecuaria (INTA), Instituto de Biotecnología, CC77, Hurlingham, Buenos Aires, Argentina; c Instituto de Horticultura, Facultad de Ciencias Agrarias, Universidad Nacional de Cuyo, Almirante Brown 500, Luján de Cuyo, Mendoza 5505, Argentina; d Instituto Nacional de Tecnología Agropecuaria (INTA) - E.E.A. La Consulta. Ex Ruta 40, km 96, La Consulta CC8, San Carlos, Mendoza 5567, Argentina

**Keywords:** Phenolic compounds, Pyruvate, Onion, Spectrophotometry, High performance liquid chromatography (HPLC)

## Abstract

We present data on absorption spectra (400–540 nm) and concentration of phenolic compounds quercetin, myricetin, kaempferol, rutin, catechin, epicatechin gallate (ECG) and epigallocatechin gallate (EGCG), in yellow, red and white onions. These data are related to the article entitled “Variability in spectrophotometric pyruvate analyses for predicting onion pungency and nutraceutical value” (Beretta et al., 2017) [1]. Given the relevance of pyruvate determinations for estimating onion pungency and functional value, it is important to identify compounds that can interfere with pyruvate determinations when using two previously published analytical procedures, namely Schwimmer and Weston (1961) (SW) [2] and Anthon and Barret (2002) (AB) [3], which are based on spectrophotometry and light-absorbance at 420 nm and 515 nm, respectively. The data presented in this article are absorption spectra for 7 onion phenolic compounds in the range 400–540 nm, which include wavelengths used by the two pyruvate analytical methods (Schwimmer and Weston, 1961; Anthon and Barret, 2002) [2,3] that were compared in our reference article (Beretta et al., 2017) [1]. Additionally, bulb content data for these 7 phenolic compounds in onion cultivars and F2 progenies with different bulb color were included to allow further analyses.

**Specifications Table**

**Table:** 

Subject area	*Biology*
More specific subject area	*Plant sciences, food chemistry*
Type of data	*Figure, graph*
How data was acquired	*High Performance Liquid Chromatography (HPLC)*
Data format	*Processed, analyzed*
Experimental factors	*Aqueous onion extracts were directly analyzed by HPLC. For quercetin and kaempferol, chemical hydrolysis was performed prior to the analysis.*
Experimental features	*Pure standards of seven phenolic compounds commonly found in onion [quercetin, myricetin, kaempferol, rutin, catechin, epicatechin gallate (ECG), and epigallocatechin gallate (ECGC)] were analyzed by HPLC. Absorption spectra in the range 400*–*540 nm and bulb contents for these seven phenolic compounds were determined in onions with different bulb color.*
Data source location	*INTA La Consulta, San Carlos, Mendoza, Argentina*
Data accessibility	*Data are within this article*

**Value of the data**

• The dataset is valuable for the identification of phenolic compounds that may interfere with onion pyruvate determinations when using the spectrophotometry-based methods of Schwimmer and Weston [2] and Anthon and Barret [3].
• Characterization of onion germplasm based on their content of seven phenolic compounds; useful for onion breeders and food technologists.
• The dataset can serve to compare flavonoids content among onion germplasm from different geographical origins.

## 1 Data

**Fig. 1:**
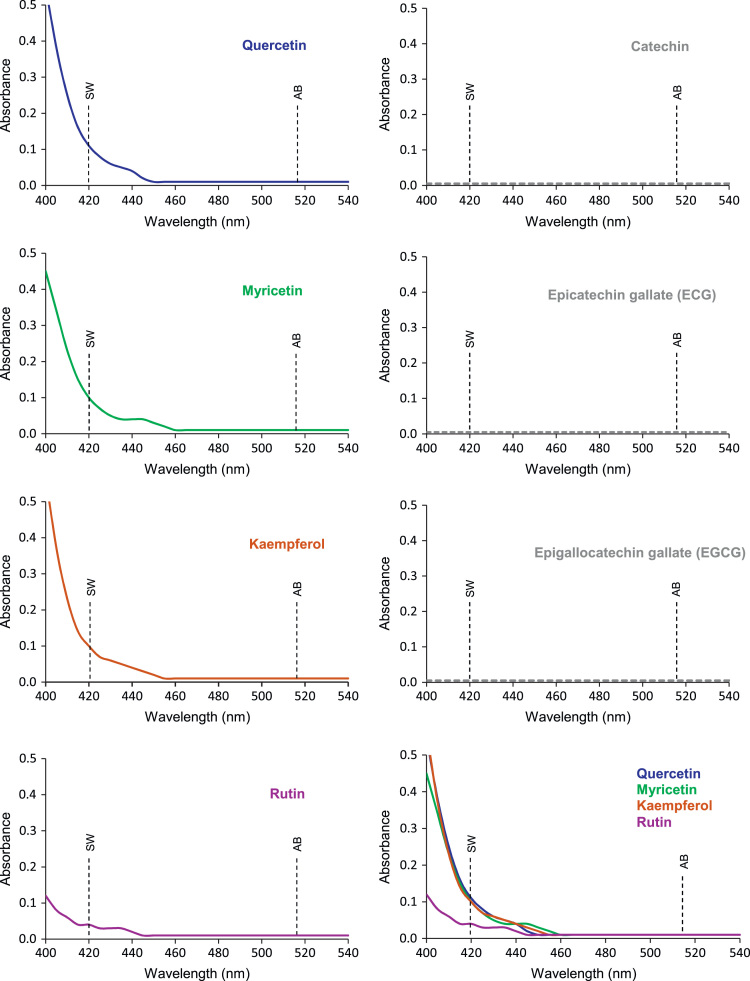
Absorption spectra (400–540 nm) for 7 standard phenolic compounds quercetin, myricetin, kaempferol, rutin, catechin, epicatechin gallate (ECG) and epigallocatechin gallate (EGCG). Dashed vertical lines indicate wavelengths used by the SW [2] and AB [3] methods for pyruvate determinations.

Fig. 1 reports light absorption spectra in the range 400–540 nm for 7 standard phenolic compounds [quercetin, myricetin, kaempferol, rutin, catechin, epicatechin gallate (ECG) and epigallocatechin gallate (EGCG)], as possible interfering compound in onion pyruvate determinations by SW and AB methods (see Beretta et al., 2017 [1]), and indicates the wavelengths used in both pyruvate methods.

**Fig. 2:**
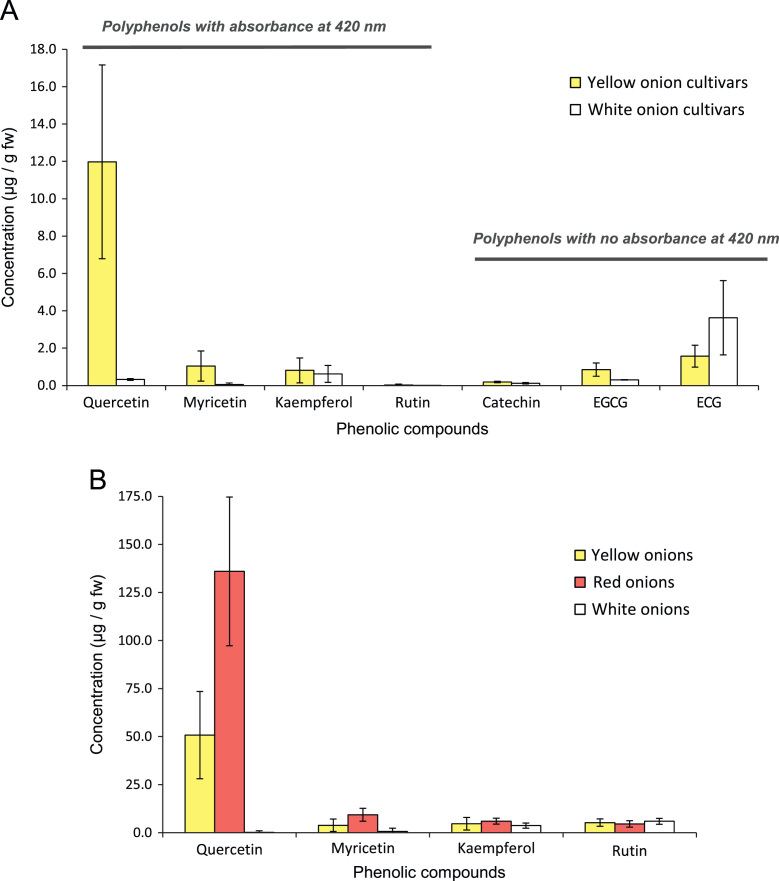
Phenolic compounds content in onion cultivars (A) and F_2_ progenies (B) with different bulb color. Only compounds that absorb light at 420 nm (i.e., compound interfering with pyruvate determinations with the SW method) were included in fig. B. Bars represent means±standard deviations.

Fig. 2 presents data on the concentration of these 7 phenolic compounds in onion cultivars (A) and onion F_2_ progenies (B) with different bulb color.

## 2 Experimental design, materials and methods

### 2.1 Reagents and samples

Pure standards (Sigma >95%) of seven phenolic compounds commonly found in onion bulbs [quercetin, myricetin, kaempferol, rutin, catechin, epicatechin gallate (ECG), and epigallocatechin gallate (ECGC)] were purchased from Sigma-Aldrich, St. Louis, USA. Methanol and acetonitrile were high performance liquid chromatography (HPLC) grade from Carlo Erba, (Carlo Erba Reagents, Milan, Italy). Trifluoroacetic acid was from J. T. Baker (Mallinckrodt Baker Inc., Phillipsburg, USA). Ultrapure water (18.2 MΩ cm) was obtained from an EasyPure II UV/UF Ultrapure Water System (Barnstead, NH, USA).

#### 2.1.1 Preparation of the standard solutions

Solutions of the authentic standards phenolic compounds were prepared in the range of 0.5–10 mg L^−1^ in methanol:water 90:10 (v/v). Equations used for quantification were obtained from a linear regression analysis (*r*^2^≥0.99).

Bulb contents for the above seven phenolic compounds were estimated, by HPLC analysis, in 5 yellow (Valcatorce, Cobriza, Valuno, Angaco, Navidena) and 3 white (Ancasti, Antartica, Refinta 20) onion cultivars (three replicates of eight bulbs per cultivar were analyzed) developed by INTA La Consulta, Argentina [4], and in 10 yellow-, 16 red- and 11 white-bulb onions from an F_2_ population developed by the Argentine onion breeding program, at INTA La Consulta. The onions were grown at the experimental field of INTA La Consulta, Argentina, using conventional agricultural practices for the crop.

### 2.2 HPLC analysis of onion phenolic compounds

Onion phenolic compounds were analyzed according to Insani et al. [5]. Briefly, onion aqueous extracts were prepared in a 1:1 volume (w/v) of distilled water using a blender (Braun MR 400 Plus, Kronberg, Germany), centrifuged and filtered through PTFE membrane filters of 0.45 μm pore size (Agilent Tech., Santa Clara, USA). For each sample, 20 μL of the filtered onion extracts were injected onto the HPLC system and myricetin, rutin, catechin, ECG, and EGCG were determined. For estimation of total quercetin and kaempferol, hydrolysis of the samples was previously performed to release the sugar molecule from the aglycon, prior to injection onto the HPLC system. For the chemical hydrolysis, an aliquot of 1/10 dilution of the onion extract in HCl 0.25 M was heated at 100 °C for 2 h in sealed vials. When samples reached ambient temperature they were filtered through PTFE and 20 μL were injected onto the HPLC system. Results were expressed as µg g^−1^ fw.

### 2.3 Chromatographic conditions

An HPLC system (Agilent 1100 series, Santa Clara, USA) equipped with a autosampler (WPALS G1367A), binary pump (G1312A), a degasser unit (G1397A), ALS Therm (G1330B), and colcom module (G1361A), was used. The analytical column was an ODS-Hypersil C18 (250×4.6 mm, 5 µm particle size). Compound detection was performed with a fluorescence detector (FLD G1321A) at 280 (excitation)-310 nm (emission) for flavan-3-ols (catechin, ECG, EGCG) and a UV–vis MWV (G1365B) at 380 nm for flavonols (quercetin, kaempferol, myricetin, rutin). The mobile phase was a linear gradient of 0.1% trifluoroacetic acid and acetonitrile: 0.1% trifluoroacetic acid 80:20 v/v. Absorbance spectra were obtained using a UV–vis Multiwavelength Spectrophotometric Detector, (Thermo Fisher Scientific Inc., Waltham, USA).
